# Study of Formulation and Process Variables for Optimization of Piroxicam Nanosuspension Using 3^2^ Factorial Design to Improve Solubility and In Vitro Bioavailability

**DOI:** 10.3390/polym15030483

**Published:** 2023-01-17

**Authors:** Yahya Alhamhoom, Sandip M. Honmane, Umme Hani, Riyaz Ali M. Osmani, Geetha Kandasamy, Rajalakshimi Vasudevan, Sharanya Paramshetti, Ravindra R. Dudhal, Namrata K. Kengar, Manoj S. Charde

**Affiliations:** 1 Department of Pharmaceutics, College of Pharmacy, King Khalid University, Abha 62529, Saudi Arabia; 2 Department of Pharmaceutics, Annasaheb Dange College of B. Pharmacy, Ashta, Shivaji University, Kolhapur 416301, Maharashtra, India; 3 Department of Pharmaceutics, JSS College of Pharmacy, JSS Academy of Higher Education and Research (JSS AHER), Mysuru 570015, Karnataka, India; 4 Department of Clinical Pharmacy, College of Pharmacy, King Khalid University, Abha 62529, Saudi Arabia; 5 Department of Pharmacology, College of Pharmacy, King Khalid University, Abha 62529, Saudi Arabia; 6 Department of Pharmaceutical Chemistry, Government College of Pharmacy, Karad, Shivaji University, Kolhapur 415124, Maharashtra, India

**Keywords:** polymers, nanosuspension, solubility, bioavailability, anti-solvent precipitation, in vitro drug dissolution

## Abstract

Piroxicam is a Biopharmaceutical Classification System (BCS) Class II drug having poor aqueous solubility and a short half-life. The rationale behind the present research was to develop a Piroxicam nanosuspension to enhance the solubility and thereby the in vitro bioavailability of the drug. Piroxicam nanosuspension (PRX NS) was prepared by an anti-solvent precipitation technique and optimized using a full-factorial design. Herein, the nanosuspension was prepared using polymer polyvinylpyrrolidone (PVP) K30^®^ and Poloxamer 188^®^ as a stabilizer to improve the solubility and in vitro bioavailability of the drug. Nine formulations were prepared based on 3^2^ full-factorial experimental designs to study the effect of the formulation variables such as concentration of poloxamer 188 (%) (X_1_) and stirring speed (rpm) (X_2_) as a process variable on the response of particle size (nm) and solubility (µg/mL). The prepared NS was characterized by phase solubility, Fourier-transform infrared (FT-IR), differential scanning calorimetry (DSC), X-ray powder diffraction (XRPD), transmission electron microscopy (TEM), particle size, zeta potential, entrapment efficiency, and percent drug release. DSC and XRPD analysis of freeze-dried NS formulation showed conversion of PRX into a less crystalline form. NS formulations showed a reduction in the size from 443 nm to 228 nm with −22.5 to −30.5 mV zeta potential and % drug entrapment of 89.76 ± 0.76. TEM analysis confirmed the size reduction at the nano level. The solubility was increased from 44 μg/mL to 87 μg/mL by altering the independent variables. The solubility of PRX NS in water was augmented by 14- to 15-fold (87.28 μg/mL) than pure PRX (6.6 μg/mL). The optimized formulation (NS9) at drug-to-stabilizer concentration exhibited a greater drug release of approximately 96.07% after 120 min as compared to the other NS formulations and pure PRX (36.78%). Thus, all these results revealed that the prepared NS formulations have improved the solubility and in vitro dissolution compared to the pure drug. Furthermore, an increase in the drug release was observed from the NS than that of the pure PRX. All these outcomes signified that the prepared PRX NS showed an increase in solubility and in vitro dissolution behavior; which subsequently would aid in attainment of enhanced bioavailability.

## 1. Introduction

There has been a growing need for alternative drug delivery systems and techniques owing to the increasing number of newly investigated drugs, increased sensitivity to clinical findings, and escalating healthcare costs. The current drug delivery system is rapidly evolving and has increased productivity. There has been significant advancement and research in delivery systems for the optimization of therapy and its cost-effectiveness. Newer pharmaceutical and biopharmaceutical product categories are accelerating the development of drug delivery technology. Often, conventional methods are unable to deliver these novel entities effectively. Thus, advanced delivery systems have therefore become increasingly important in today’s world. Most of the recently developed drug molecules have poor solubility, which can lead to significant formulation issues and poor bioavailability.

Solubility is one of the key parameters to achieve the desired concentration of drug in the systemic circulation necessary for achieving the required pharmacological response. Following oral administration, poorly water-soluble drugs require high dosages to attain therapeutic plasma concentration. Water insolubility is one of the central elements which restrict the usage of many potential drug moieties and other active compounds. Because of this, the bioavailability of the drug is less and it fails to reach the site of action [1]. Solubility is closely associated with bioavailability. It greatly increases the bioavailability of the dosage form. Only highly soluble drug molecules can cross the cell membrane and show their desired therapeutic effect by reaching the site of action [2,3]. In vitro dissolution of the drug is related to its in vivo bioavailability [2]. The primary strategy in this study domain is enhancing the solubility of BCS class II drugs. Solubility of the drugs can be improved by a variety of techniques, such as particle size reduction, micro-emulsion, micellar solubilization, solvent deposition, Super Critical Fluid (SCF) process, solid dispersion, nanosuspension (NS), cryogenic techniques, inclusion complex formation-based techniques, hydrotropy, co-crystallization, complexation, liquid-solid system, etc. [2,4].

The majority of the aforementioned solubility enhancement methods, including NS, may be utilized to make drugs more soluble. In developing an ideal formulation, several factors, such as stability at various temperatures, solubility, compatibility of the solvent and excipients, and photostability, are essential. Thus, the present study aimed in developing NS to resolve issues relating to low solubility and poor bioavailability [5]. A drug that is weakly water soluble and free of any matrix material can be utilized to develop NS [6]. NS improves medication safety and effectiveness by resolving the challenges of low solubility and bioavailability as well as by changing the pharmacokinetics of the drug [7]. NSs are colloidal dispersions having surfactant-stabilized drug particles that are nanoscale in size [4,8]. Pure drug particles are dispersed in the aqueous medium to create a biphasic system known as an NS. The suspended particle has a diameter of less than 1 µm. The increase in surface area and saturation solubility of the drug particles results from the reduction in drug particle size, which accelerates the dissolution. The increased vapor pressure of the particles leads to increased saturation solubility and solution velocity of the nanoparticles. Because of these properties, NS is the best technique for enhancing the water solubility and dissolution rate of drugs [9,10]. The particle size distribution, surface charge, crystalline state, dissolution rate, and saturation solubility are the main characteristics of oral NSs. A zeta potential of at least ±30 mV is needed for an electrostatically stabilized NS to be physically stable. A general guideline line of ±20 mV will be sufficient in the case of a combined steric and electrostatic stabilization. For pharmaceuticals that exist in several polymorphs, the crystallinity of the NS is crucial [11]. 

The drug Piroxicam(PRX) [4-Hydroxy-2-methyl-N-(2-pyridinyl)-2H-1, 2-benzothiazine-3-carboxamide 1,1-dioxide] belongs to the class of anti-inflammatory drugs. PRX demonstrates prolonged and delayed oral absorption [12]. It is a highly protein-bound medication that is slowly removed from the body, increasing the half-life to up to 36 to 86 h. It has various side effects such as diarrhea, constipation, headache, dizziness, and ringing in the ears. Although it has a variety of side effects, PRX has a stronger pharmacological efficacy because it is a potent anti-inflammatory drug [12]. In the US, PRX is approved for the treatment of rheumatoid arthritis and osteoarthritis [13]. PRX is a BCS class II medication that has high permeability and low solubility [2]. It exhibits a slow and gradual absorption when taken orally and is proven to be ulcerogenic. Therefore, the need of the hour is to develop novel formulations that would accelerate its absorption in the GI tract and might give quick relief from rheumatoid arthritis and osteoarthritis with a reduction in its dose and dose-dependent side effects. The NS development approach has viable potential for enhancing solubility of poorly soluble drugs and is also cost effective, simple and robust. PRX in the form of NS is a practically executable and promising way to mitigate this problem. Hence, the objective of the current study was to investigate the effects of a formulation variable (polymer concentration) and process parameters (stirring speed and time) on the NS formulation of PRX with the intent of attainment of improved solubility and in vitro bioavailability. In the present study, NS has been chosen as an approach to enhance the solubility, dissolution release and in vitro bioavailability of PRX. Differential scanning calorimetry (DSC), X-ray powder diffraction (XRPD), and transmission electron microscopy (TEM) analysis were performed to determine the solid-state properties of the drug in physical mixtures and NSs in comparison with the free PRX.

## 2. Materials and Methods

### 2.1. Materials

PRX was procured from Zydus Cadila Healthcare Ltd., Goa, India. The following chemicals were obtained commercially: PVP K30^®^, Poloxamer 188^®^, Methanol, Hydrochloric Acid and Dichloromethane (Loba Chemie, Mumbai, India). All the other chemicals, solvents and reagents used were of analytical grade and were stored and used as per the supplier’s instructions.

### 2.2. Methods

#### 2.2.1. Pre-Formulation Study

Pre-formulation studies were carried out to determine the characteristics of the drug and excipients, particularly on the physicochemical, physicomechanical, and biopharmaceutical aspects [14].

#### 2.2.2. Organoleptic Evaluation

The drug sample was evaluated for organoleptic properties. The organoleptic evaluation was conducted by observing the appearance, color, and odor of the drug sample.

#### 2.2.3. Melting Point

Using a micro-controlled based melting point apparatus (SMP10/1, Stuart, UK), the melting point of the drug was determined. The drug sample was inserted into a capillary tube with one end closed. The capillary tube was inserted into a silicone oil bath, which was heated with the help of an electrical heating coil in a controlled manner. The temperature at which the drug sample started melting was noted as the melting point temperature. The average of triplicate readings was noted and compared with the literature value.

#### 2.2.4. Ultraviolet-Visible (UV-Visible) Spectrophotometry

The absorbance maxima (λ_max_) of PRX were determined in various solvents such as methanol, methanolic HCl, phosphate buffer solution (PBS) pH 6.8, and PBS pH 7.4 in the range of 200–400 nm by using a UV-Visible double beam spectrophotometer (Shimadzu 1800, Tokyo, Japan) [14].

#### 2.2.5. Determination of Calibration Curve

The calibration curve of PRX has been investigated in different solvents such as methanol, methanolic HCl, PBS pH 6.8, and PBS pH 7.4 at specific wavelengths [14].

#### 2.2.6. Solubility of Drug

Drug solubility was assessed in a variety of solvents, including distilled water, PBS pH 7.4, and methanol. In order to create saturated solutions, the excess drug was added to the vehicles, which were then shaken continuously for 48 h at a temperature of 25 ± 0.5 °C. The solutions were filtered, diluted and analyzed using UV spectrophotometry (Shimadzu 1800, Japan) [14].

#### 2.2.7. Fourier-Transform Infrared (FT-IR) Spectroscopy 

Drug-excipient compatibility was confirmed by FT-IR spectroscopy (Bruker Alpha II). The spectra were obtained using the KBr pellet method within the range of 4000 cm^−1^ to 400 cm^−1^. Briefly, the pellets were prepared with KBr in a ratio of 1:100 and force was applied for several minutes to obtain uniform thin pellets. These pellets were placed in between two plates in a sample holder and scanned. The absorbance was plotted against their corresponding wavenumber [12].

#### 2.2.8. Preparation of NS

**Preparation of drug solution:** the required amount of the drug was dissolved in 4 mL dichloromethane to obtain a clear solution [15].

**Preparation of polymer solution:** the required amounts of Poloxamer 188 and PVP K30 were dissolved in water. Using a mechanical stirrer (Remi RQT 124 AD, Mumbai, India), the polymer solution was homogenized at 1000–1200 rpm. Then, the drug solution was added dropwise into the polymer solution using a syringe and stirred continuously followed by sonication for 20 min. From the preliminary study, based on complete mixing and the optimal particle size, stirring time was optimized at 15 min [15,16].

### 2.3. Optimization of Formulation

A randomized, 3^2^ full factorial design with two factors and three levels was employed to systematically study the nanosuspension formulation (Table 1). A total of nine experimental trials were performed at all possible combinations. The concentration of stabilizer and stirring speed were identified as the independent variables based on the experiments conducted during the optimization, which were altered at three different levels, i.e., low, medium, and high. Solubility (µg/mL) and particle size (nm) were considered as dependent variables (responses) The response variables used were solubility and particle size (nm). The development and evaluation of the statistical experimental design were accomplished by utilizing the Design-Expert 8.0 software (Stat-Ease Inc., Minneapolis, USA). The effect of two independent variables, stabilizer concentration (X_1_) and stirring speed (X_2_), on the response (Y) was studied.

**Formulation optimization using the desirability function:** using Design-Expert 8.0 software (Stat-Ease Inc., USA), various response surface methodology (RSM) computations were carried out for the current optimization research. All of the response variables were developed in a linear model with quadratic terms. Additionally, using the Design-Expert software output files, linear plots and 3D graphs were developed. Analysis of variance (ANOVA) was used to determine the importance of these characteristics on the variables.

The optimization process employed the desirability function once the mathematical model had been fitted. The results were combined to find a product with the desired properties during formulation optimization. The desirability function predicts the ideal values for the independent variables by combining all the results into one variable. The least desirable value for the replies is represented by a desirability value of 0, while the most desirable value is represented by a desirability value of 1.

#### 2.3.1. Freeze-Drying of Optimized Formulation

The optimized batch (NS9) was lyophilized using mannitol as a cryoprotectant for 24 h under controlled conditions to obtain dry powder using a laboratory-scale lyophilizer (Alpha 1–2 L Dplus, Martin Christ Gefriertrocknungsanlagen GmbH, Germany). The product was stored in an airtight container until further characterization [17].

#### 2.3.2. Characterization of NS

**Determination of phase solubility of NS:** for the determination of the phase solubility of PRX, different concentrations of Poloxamer 188 were prepared. Briefly, an excess amount of the drug (1 g) was added to each of the 250 mL flasks containing 25 mL of stabilizer poloxamer 188 having three distinct concentrations (0.1%, 0.3%, and 0.5%) and at different speeds. The flasks were properly sealed and agitated for 48 h at 37 °C at 100 rpm in an orbital shaker cum incubator (Orbit™ 1000 multipurpose digital shaker). For the establishment of the equilibrium, they were kept in the incubator for a further 24 h. Five (5) mL of the supernatant was filtered and appropriately diluted, and the amount of drug in the filtrate was evaluated photometrically using a UV-Visible spectrophotometer (Shimadzu 1800, Tokyo, Japan) at 354 nm [18].

**Particle size, polydispersity index and zeta potential analysis:** by using the dynamic light scattering (DLS) technique, the mean particle size, polydispersity index and zeta potential were determined using a particle size analyzer (Horiba Scientific SZ-100). Freshly prepared NS was diluted 100 times with distilled water and analyzed [17,19].

**Determination of Entrapment Efficiency (EE):** entrapment efficiency (EE) of the optimized formulation was determined by quantitatively estimating the amount of drug loaded into the NS. The NS formulation was ultracentrifuged (Optima KE-90-IVD, Beckman Coulter, Pasadena, CA, USA) for 20 min; the resulting supernatant was diluted sufficiently with methanol for subsequent UV-spectrophotometric analysis at 334 nm [20].

(1) $$\%\;\mathrm{EE}=\frac{\mathrm{Total}\;\mathrm{amount}\;\mathrm{of}\;\mathrm{drug}\;-\;\mathrm{Free}\;\mathrm{drug}\;}{\mathrm{Total}\;\mathrm{amount}\;\mathrm{of}\;\mathrm{drug}}\times 100$$

**Transmission Electron Microscopy (TEM):** the size and shape of the optimized NS were evaluated by means of TEM (H-7500, Hitachi, Tokyo, Japan).

**Differential Scanning Calorimetry (DSC) analysis:** a differential scanning calorimeter (Mettler-Toledo DSC 821e, Columbus, OH, USA), was used to carry out the thermal analysis for confirming the compatibility between the drug and the excipient. The thermal behavior of the drug and the optimized formulation was investigated via DSC analysis. Briefly, the samples were weighed accurately and sealed hermetically in aluminum pans and crimped and were heated from 25 to 250 °C at a heating rate of 10 °C/min. Throughout the measurement, nitrogen gas was purged over the sample cell with a flow rate of 50 mL/min [21].

**X-ray Powder Diffraction (XRPD):** XRPD is a crucial method used to determine the crystalline or amorphous nature of the sample. Using a powder X-ray diffractometer (AXS D8 Advances, Bruker Ltd., Germany), diffractograms of the pure drug and NS formulation were obtained with tube anode Cr over the interval of 10–70°/2θ using copper as an X-ray target, with 1.54 Å wavelength [21,22].

**In vitro dissolution study:** a USP Type II dissolution testing paddle apparatus was used for determining the in vitro dissolution. An amount of sample which is equivalent to 10 mg of PRX was added to the glass jar of apparatus containing 900 mL of PBS pH 6.8 maintained at a temperature of 37 °C [18,23]. Then, for 2 h, the paddle was rotated at 75 rpm. Three (3) mL of sample was taken out at predetermined intervals, filtered, and properly diluted. The concentration of the drug dissolved in the medium was then determined with the help of a UV spectrophotometer (Shimadzu 1800, Tokyo, Japan). To maintain a constant volume of a medium during the dissolution, 3 mL of fresh medium was always replaced in the glass jar.

## 3. Result and Discussion

### 3.1. Pre-formulation Study

#### 3.1.1. Organoleptic Properties

For identifying the compound, the organoleptic features of the drug sample, such as appearance, color, and odor were examined. Table 2 displays outcomes that adhere to the standards set out in the existing literature.

#### 3.1.2. Melting Point

The melting point of PRX was determined using the capillary method. It has been noted in the range of 198–200 °C. The experimental values are in good agreement with the criteria in the published literature [13].

#### 3.1.3. Determination of Maximum Wavelength (λ_max_)

**Figure 1:**
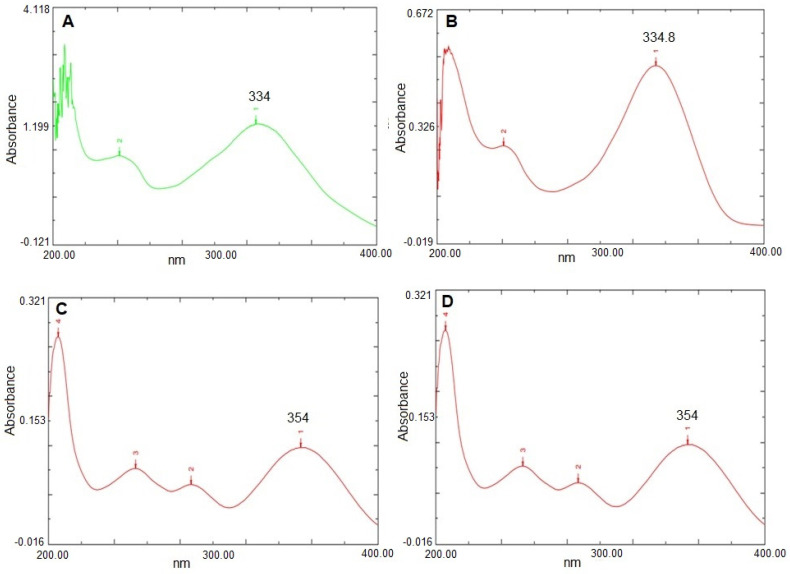
UV spectra depicting λ_max_ of PRX in methanol (**A**), methanolic HCl (**B**), PBS pH 6.8 (**C**) and PBS pH 7.4 (**D**).

The absorption maxima (λ_max_) of PRX in the methanol, methanolic HCl, PBS pH 7.4, and PBS pH 6.8 is represented in Figure 1 and the same has been depicted in Table 3.

#### 3.1.4. Preparation of Calibration Curve

The calibration curve of PRX was plotted in methanol, methanolic HCl, PBS pH 6.8, and PBS pH 7.4. It showed the linear relationship in the concentration range of 2-10 µg/mL. The R^2^ values were obtained above 0.99.

#### 3.1.5. Solubility of Drug

In contrast to being poorly soluble in water, PRX readily dissolves in methanol, ethanol, and dichloromethane, among other organic solvents. PRX solubility was investigated in a variety of solvents including water, PBS pH 7.4, and methanol. The investigation shows that the drug was freely soluble in methanol, soluble in PBS pH 7.4, and very slightly soluble in water (Table 4).

#### 3.1.6. FT-IR Studies

**Figure 2:**
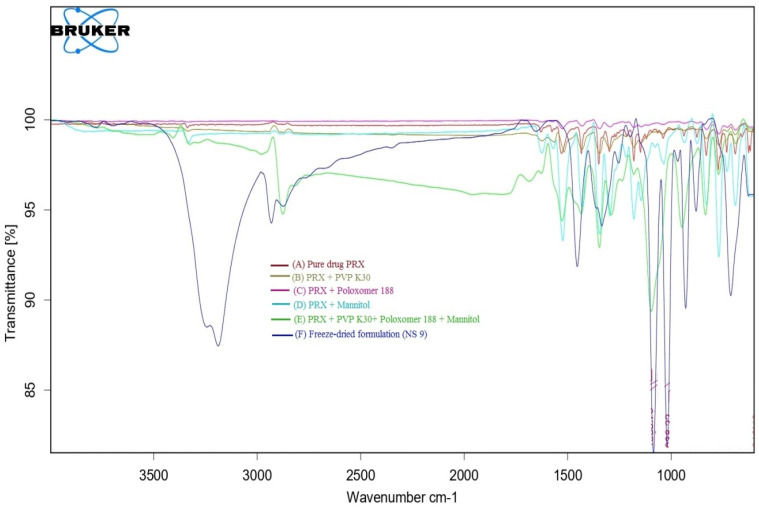
Overlain FT-IR spectra of PRX (A), physical mixture of PRX and PVP K30^®^ (B), physical mixture of PRX and Poloxamer 188^®^ (C), physical mixture of PRX and mannitol (D), physical mixture of PRX, PVP K30^®^, Poloxamer 188^®^ and mannitol (E), and freeze-dried optimized formulation (NS9) (F).

FT-IR studies predict the possible interaction between the drug and the excipients. FT-IR spectra (Figure 2) of pure PRX, its physical mixture and optimized formulation depicted all the characteristic IR peaks corresponding to their functional groups as reported in the literature. (Table 5).

All the characteristic peaks were observed in the IR spectra of pure PRX and in the physical mixture of PRX, poloxamer 188^®^, PVP K 30^®^, and mannitol. This indicates that there is no interaction between the PRX and excipients.

### 3.2. Preparation of NS

PRX NS was prepared by the antisolvent-precipitation technique. The aqueous phase containing suitable polymer and stabilizer (PVP K30^®^ and Poloxamer 188^®^) was used as the antisolvent and dichloromethane was used as a solvent.

#### 3.2.1. Full Factorial Design

The experimental design shown in Table 1 is a factorial design for two variables at three different levels using −1, 0, and +1 corresponding to a 3^2^ full factorial design. It is possible to evaluate the impact of distinct variables (primary effects) and their second-order effects using this efficient second-order experimental design with a small number of tests. Further, this design has the advantage of calculating the quadratic response surface, which is not estimable using a factorial design at two levels. A full factorial design was used to carefully evaluate the variables.

Data from the experimental runs were subjected to regression and graphical analysis, which produced the equations in Table 6 with F ratios that were statistically significant (*p* < 0.05) and Adj-R^2^ values in the range of 0.7–1. The data were well-fit by these model equations.

The significance of the effect on particle size (Y_1_) was established using ANOVA and the linear regression equation fit to the data was:

Y_1_ = 326.56 − 70.50 × X_1_ − 41.67 × X_2_ (2)

As the stabilizer concentration and stirring speed rise, respectively, the negative sign at coefficients X_1_ and X_2_ indicate a reduction in particle size. Because super-saturation occurs with increasing drug concentration in the aqueous phase and leads to fast precipitation on diffusion, particle size was reduced. In order to prevent agglomeration, the drug particles were reduced in size up to nanosized ranges and well-protected by a stabilizer. Moreover, considerable stabilizer upholds Oswald’s ripening while too little stabilizer causes agglomeration or aggregation and increases particle size (a phenomenon in which smaller particles get smaller due to more solubility, and larger particles become larger through re-precipitation of small particles on it). The stabilizer concentration was found to be ideal between 0.2 and 0.6 percent. The same observation has been reported by Ahire et al. [24]. The increase in the stirring speed also results in the reduction in the particle size.

The effect on solubility (Y_2_) was established to be meaningful by ANOVA and the linear regression equation fit to the data was:

Y_2_ = 67.23 + 11.80 × X_1_ + 6.36 × X_2_ (3)

**Figure 3:**
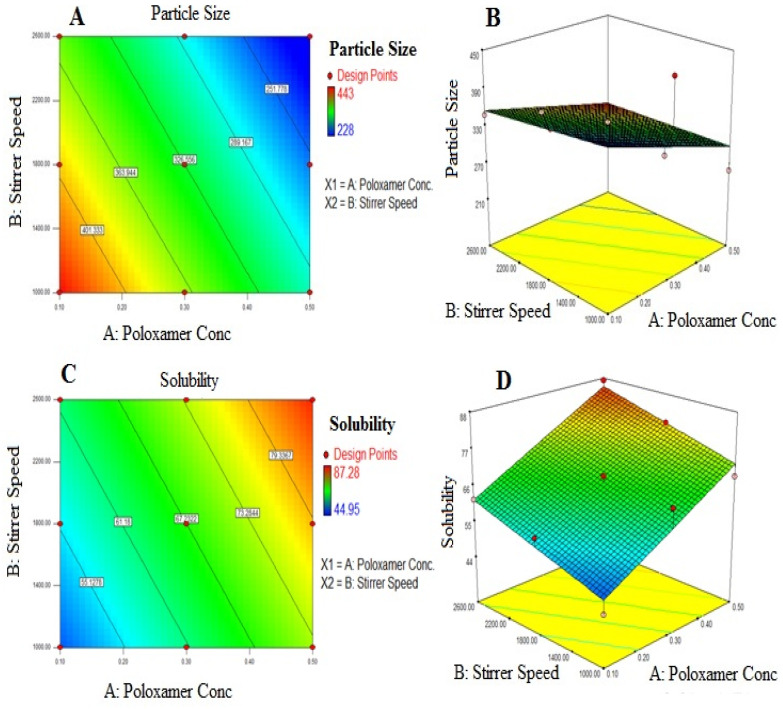
Linear and 3D response surface plot for independent variable’s effects on the response particle size (**A**,**B**), respectively, and on response solubility (**C**,**D**), respectively.

Poor wetting of the drug surface is a sign of hydrophobicity, which contributes to low solubility. As a result of this, the particles agglomerate instead of dispersing. Because of the increased surface area brought on by the reduction in particle size, the drug’s solubility in the NS was enhanced shown in Figure 3. The increase in solubility (Y_2_) is predicted by the positive coefficient of X_1_ and X_2_ as the stabilizer concentration and stirring speed, respectively.

#### 3.2.2. Formulation Optimization Using the Desirability Function

The pharmaceutical formulation is optimized by studying the effects of different levels of variables on the responses. During the optimization process, all measured responses that can have an impact on the product’s quality were taken into account. Considering the effect of stabilizer concentration (0.5%) and stirring speed of 2600 rpm and manipulating data from Table 6 it was observed that the optimized formulation (NS9) showed a decrease in particle size (228 nm) and high solubility (87.28 µg/mL).

### 3.3. Characterization of NS

#### 3.3.1. Phase Solubility of NS

The initial solubility of PRX in distilled water was found to be 6.6 µg/mL. The solubility study of NS was performed at different concentrations of stabilizer, i.e., poloxamer 188^®^ and at a different speed. The results showed an increase in the solubility of PRX in the NS formulations (Table 7).

#### 3.3.2. Particle Size and Zeta Potential

**Figure 4:**
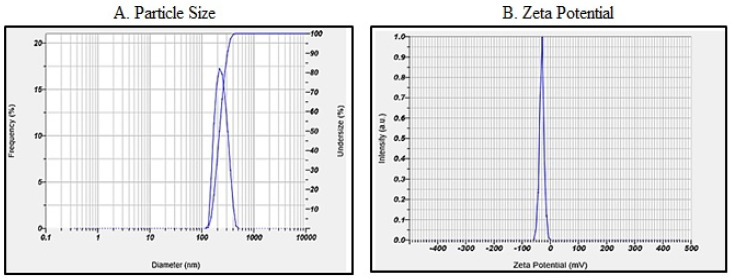
Particle size distribution curve (**A**) and zeta potential peak (**B**) of the optimized formulation NS9.

The particle size in the NS formulation was found to be decreasing with an increase in the poloxamer 188^®^ concentrations and an increase in the speed as shown in Table 7. The particle size and zeta potential of the optimized formulation are shown in Figure 4.

Entrapment efficiency.

The amount of non-capsulated PRX was determined by an indirect method. The entrapment efficiency of NSs is shown in Table 7. The optimized formulation (NS9) exhibited the highest drug entrapment, i.e., 89.76 ± 0.76%.

#### 3.3.3. TEM Study

**Figure 5:**
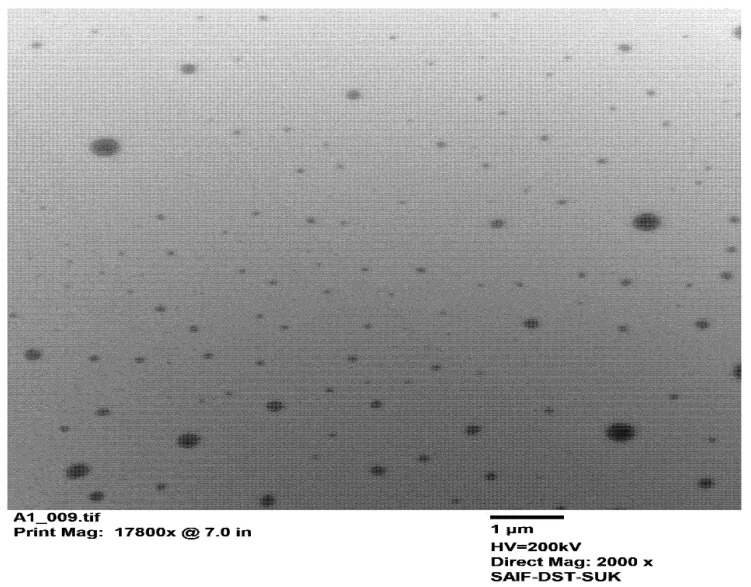
TEM micrograph of optimized PRX NS formulation (NS9).

TEM analysis was used to depict the morphology of suspended PRX nanoparticles, and the resulting TEM micrograph of the optimized NS is shown in Figure 5. TEM analysis revealed that the suspended nanoparticles were roughly spherical or irregular in shape with uniform distribution; these findings were in good agreement with the particle size analysis outcomes of the optimized formulation NS9 suggesting an average particle size of 228 nm.

#### 3.3.4. DSC STUDY

**Figure 6:**
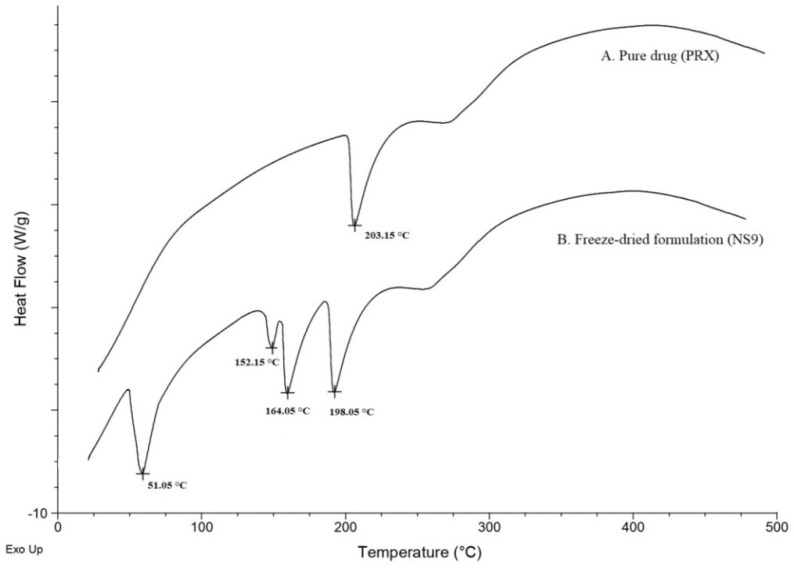
Overlain DSC thermograms of the pure drug (PRX) (A) and freeze-dried formulation (NS9) (B).

DSC was used to examine the thermal behavior of the drug and nanoparticles. The pure drug shows a sharp endothermic peak, which corresponds to its melting point, which was observed at 203.15 °C. However, a significant shift in the melting peak of the pure drug (PRX) in the nanoformulation was observed at 198.05 °C, indicating a reduction in crystallinity compared to pure PRX (Figure 6). This indicated the change in the crystalline nature of PRX during the preparation of nanosuspension. The shift also may be due to the presence of stabilizers (Poloxamer 188^®^ at 51.05 °C and PVP K30^®^ at 152.15 °C) in the formulation when compared with the pure drug. A sharp endothermic peak at 164.05 °C represents the melting of mannitol used as a cryoprotectant in the formulation. The conclusions drawn from the DSC analysis were in good agreement with the noted FT-IR outcomes and were further validated using the results of the XRPD analysis. 

#### 3.3.5. XRPD Analysis

**Figure 7:**
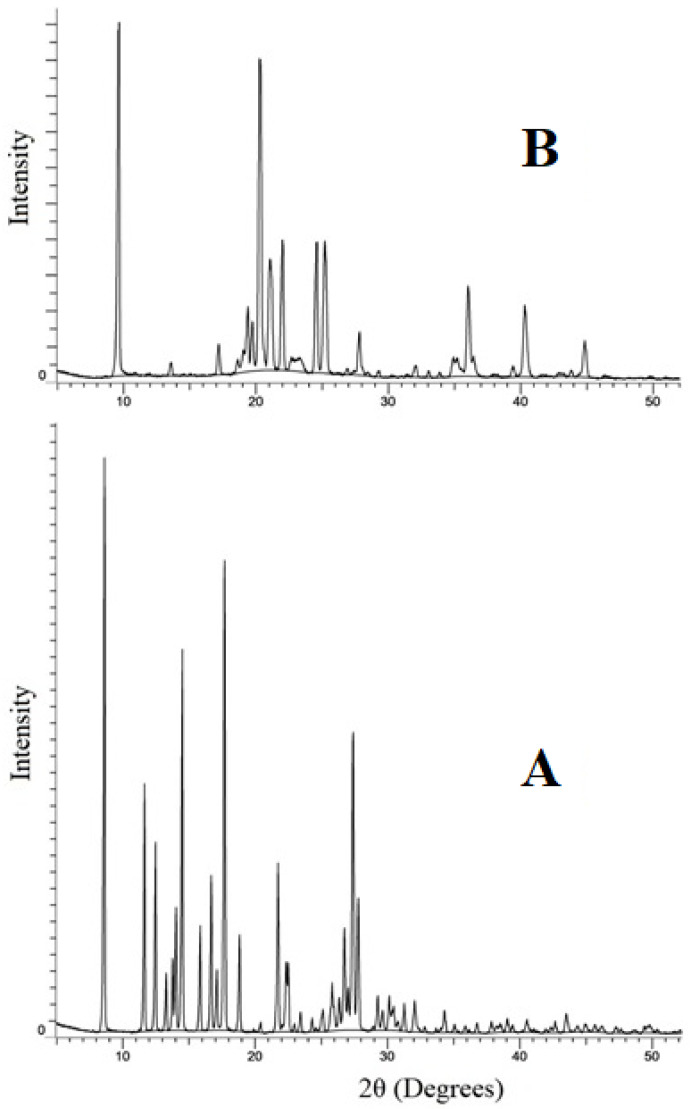
XRPD diffractograms of (**A**) pure drug PRX and (**B**) freeze-dried optimized formulation NS9.

To characterize the crystalline nature of PRX within the NS and to evaluate the mode of interaction between PRX and NS, XRPD data of pure PRX and freeze-dried formulation (NS9) samples were acquired (Figure 7). Typical diffraction peaks at 8.62°, 11.65°, 12.49°, 13.28°, 14.51°, 15.86°, 16.70°, 17.71°, 18.85°, 21.74°, and 27.80° were used to identify the crystalline nature of PRX. The pure PRX exhibits an intense crystalline peak between 10° and 30°. However, the peaks in the NS9 formulation were less intense; indicating a decrease in crystallinity. Additionally, the peaks at 9.10°, 20.22°, 36.42°, 40.80°, and 45.10° are the peaks of D-mannitol used in freeze drying which was absent in pure drug (PRX). Similar kinds of results were reported in previous research studies in the literature [25,26].

#### 3.3.6. In Vitro Dissolution Study

**Figure 8:**
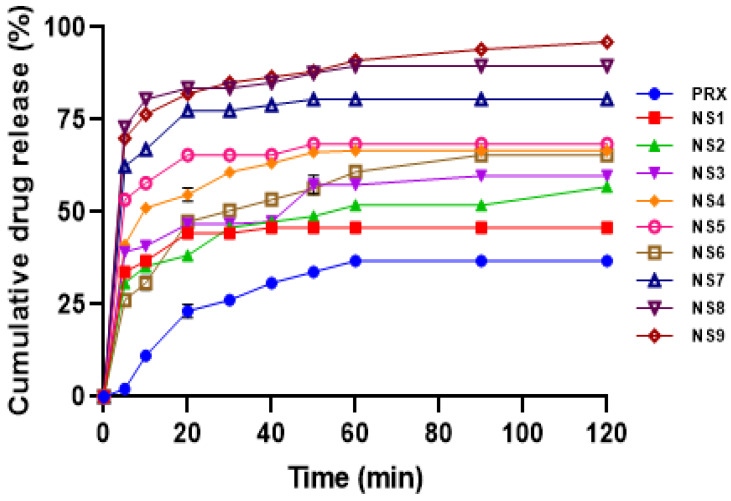
In vitro drug release profiles of prepared PRX NS formulations.

The in vitro dissolution profile of all the NS formulations (Figure 8) revealed that the PRX was released significantly faster than that of the pure drug. The optimized formulation (NS9) exhibits a greater drug release of approximately 96.07% after 120 min as compared to the other NS formulations and pure PRX (36.78%). According to the dissolution profile, all formulations exhibit burst release, which may be caused by the solubilized drug present in the NS. PRX was more easily dissolved by the formulation when the stabilizer, Poloxamer 188^®^, was used in higher concentrations and stirred at higher rpm. This improvement can be due to the ability of PVP K30^®^ and stabilizer Poloxamer 188^®^ to form a complex with PRX altering its crystalline nature, which leads to an increase in the solubility and dissolution rate of the PRX in NS than pure PRX. Dissolution efficiency is the area under the dissolution curve within a given range of time. The amount of drug dissolved and the time of solution in contact with the region of absorption, i.e., the GI tract, directly correlate with drug absorption, which increases drug bioavailability.

#### 3.3.7. Mathematical Modeling Studies

The R^2^ values from fitting the experimental model’s in vitro dissolution data into the various release kinetic models are shown in Table 8. Pure PRX drug release data were extremely well-described by the Higuchi model, whereas the Korsmeyer-Peppas model was found to best fit for NS. The Korsmeyer-Peppas model was the most accurate model for describing the release mechanism for all formulations, while all other models were the least accurate.

The highest R^2^ value noted for the Korsmeyer-Peppas model (0.9929) established it to be the best-fitting model and the release exponent (*n*) value of NS was below 0.5 which indicated that NS had followed Quasi-Fickian release kinetics [27]. This Fickian diffusion corroborates to the transport of water via the diffusion process which was driven by the concentration gradient, i.e., drug release from high concentration to low concentration.

## 4. Conclusions and Future Scope

Nano-sizing is the finest method to improve the solubility and dissolution rate of poorly water-soluble drugs. The anti-solvent precipitation method implied in current research vocation is a cost-effective and simple approach for formulating NS. This research work was aimed at optimizing the NS formulation of PRX by varying concentrations of stabilizers and stirring speed. Optimization was successfully achieved implying 3^2^ full factorial design as a Quality by Design (QbD) approach. The obtained 3D surface response analysis results and plots revealed that increasing the stabilizer concentration and stirring speed had resulted in the reduction in particle size and increase in solubility. Different NS batches have depicted a reduction in the particle size from 443 nm to 228 nm and the solubility has increased from 44 μg/mL to 87 μg/mL over changing the independent variables. From all the noted results, it was concluded that the solubility of PRX in water was enhanced by 14- to 15-fold (87.28 μg/mL) than that of the pure PRX (6.6 μg/mL). The optimized NS formulation (NS9) exhibited a greater in vitro drug release of approximately 96.07% post-120 min with respect to the other NS formulations and pure PRX (36.78%). Moreover, the in vitro drug release data from the PRX NS was fitted into diverse mathematical models to predict the drug release mechanism. The release kinetics outcomes established that release profile best fits into the Korsmeyer-Peppas model suggesting the Fickian diffusion as drug release mechanism. Furthermore, from the DSC and XRPD analysis data, it was evident that the PRX was converted into a less crystalline form when formulated into NS, which could be a valid justification for the enhanced PRX solubility. In a nutshell, from all the noted results, it was evident that the NS formulations had improved the solubility and in vitro dissolution of PRX quite significantly in comparison with the pure drug. Hence, from the in vitro-in vivo correlation (IVIVC) point of view, we conclude that prepared and optimized NS formulation would also lead to an increase in the bioavailability of PRX. However, to validate the in vivo performance and to establish the in vivo pharmacokinetic parameters in detail, a multicentric, randomized in vivo pharmacokinetic study should be undertaken in near future. To conclude, the present research outcomes have clearly indicated that NS formulation approach could be a potential alternative to the conventional methods of solubility as well as bioavailability enhancement and need to be explored much for the diverse promising existing and newly developed BCS Class II drugs. 

## Figures and Tables

**Table 1:** 3^2^ full factorial design with composition and independent variables.

Formulation Code	Composition			Speed (rpm)	Total Volume (mL)
	PRX (mg)	PVP K30^®^ (mg)	Poloxamer 188^®^ (%*w*/*v*)		
NS1	50	50	0.1 (−1)	1000 (−1)	100
NS2	50	50	0.1 (−1)	1800 (0)	100
NS3	50	50	0.1 (−1)	2600 (+1)	100
NS4	50	50	0.3 (0)	1000 (−1)	100
NS5	50	50	0.3 (0)	1800 (0)	100
NS6	50	50	0.3 (0)	2600 (+1)	100
NS7	50	50	0.5 (+1)	1000 (−1)	100
NS8	50	50	0.5 (+1)	1800 (0)	100
NS9	50	50	0.5 (+1)	2600 (+1)	100

−1: low level, 0: medium level, and +1: high level of independent variables.

**Table 2:** Organoleptic properties of PRX.

Sr. No.	Identification Test	Observation	Standard
1	Appearance	Off-white powder	Off-white to light tan or light-yellow powder
2	Odor	Odorless	Odorless
3	Color	White	Off-white

**Table 3:** λ_max_ of PRX in different solvents.

Solvent	λ_max_ (nm)	
	Observed	Standard [12]
Methanol	334	334
Methanolic HCl	334.8	334
PBS pH 7.4	354	354
PBS pH 6.8	354	354

**Table 4:** Solubility of the drug in different solvents.

Sr. No.	Solvent	Reference Solubility (mg/mL)	Observed Solubility (mg/mL)
1	Methanol	5.0	4.44
2	PBS pH 7.4	0.592	0.6606
3	Water	0.0076	0.0066

**Table 5:** Interpretation data of the FT-IR spectral analysis.

Characteristics Functional Groups.	Standard Range (cm^−1^)	Observed Peak for Pure PRX (cm^−1^)	Observed Peak for NS Physical Mixture (cm^−1^)
–OH and –NH Stretching	3650–3300	3337.34	3337.72
Aromatic –C=C–H	3300–2700	2374.30	2878.27
C=O Stretching	1850–1680	1628.71	1628.93
Aromatic –C=C–	1680–1450	1575.84	1575.95
Ar–NH	1360–1250	1349.93	1342.49
N-CH_3_ Stretching	1220–1050	1148.62	1147.59
–SO_2_-N= Group	1070–1050	1065.53	1059.18
o-disubstituted phenyl	750	772.14	771.90

**Table 6:** Results of statistical analysis of the 3^2^ full factorial experimental design.

Response	Sources			
	Model *p* Value	R^2^	Adjusted R^2^	Predicted R^2^
Particle Size	0.0053	0.8255	0.7673	0.5975
Solubility	0.0005	0.9217	0.8956	0.8126

**Table 7:** Particle size, PI, zeta potential, PDE, and solubility of the NSs.

Formulation Code	Particle Size (nm)	PI	Zeta Potential (mV)	* PDE ± SD	* Solubility ± SD (µg/mL)
NS 1	412	0.706	−22.5	89 ± 0.14	44.95 ± 0.63
NS 2	389	0.713	−24.7	89.48 ± 1.02	57.86 ± 1.21
NS 3	342	0.794	−25.6	89.4 ± 0.21	61.74 ± 0.63
NS 4	443	0.705	−29	88.42 ± 0.32	66.6 ± 1.04
NS 5	334	0.615	−35.9	90.54 ± 0.80	69.65 ± 1.96
NS 6	287.7	0.722	−34.2	88.87 ± 0.95	68.96 ± 1.24
NS 7	258	0.703	−26.4	88.44 ± 1.05	68.96 ± 0.41
NS 8	240	0.566	−28.2	89.41 ± 0.75	79.09 ± 0.24
NS 9	228	0.458	−30.5	89.76 ± 0.76	87.28 ± 0.24

* All values are expressed as mean ± SD (*n* = 3).

**Table 8:** Kinetic profiles of in vitro drug release of optimized PRX NS (NS9).

Dissolution Medium	Zero Order	First Order	Higuchi Model	Hixson-Crowell	Korsmeyer–Peppas		Diffusion Mechanism
	R^2^				R^2^	Release Exponent (*n*)	
PBS pH6.8	0.82	0.9797	0.9413	0.9432	0.9929	0.098	Quasi Fickian Diffusion

## Data Availability

The data in support of the findings of this study will be available from the corresponding author upon request.
